# Supplementary material for: Physiological significance of proteolytic processing of Reelin revealed by cleavage-resistant Reelin knock-in mice
Source: Sci Rep. 2020 Mar 11;10:4471. doi: 10.1038/s41598-020-61380-w (PMC7066138; doi:10.1038/s41598-020-61380-w)
Supplement: Supplementary file 1 — Supplementary Information. [file 41598_2020_61380_MOESM1_ESM.pdf]

Supplementary Information for :

Physiological significance of proteolytic processing of Reelin revealed by cleavage-resistant Reelin knock-in mice

Eisuke Okugawa, Himari Ogino, Tomofumi Shigenobu, Yuko Yamakage, Hitomi Tsuiji, Hisashi Oishi, Takao Kohno, and Mitsuharu Hattori

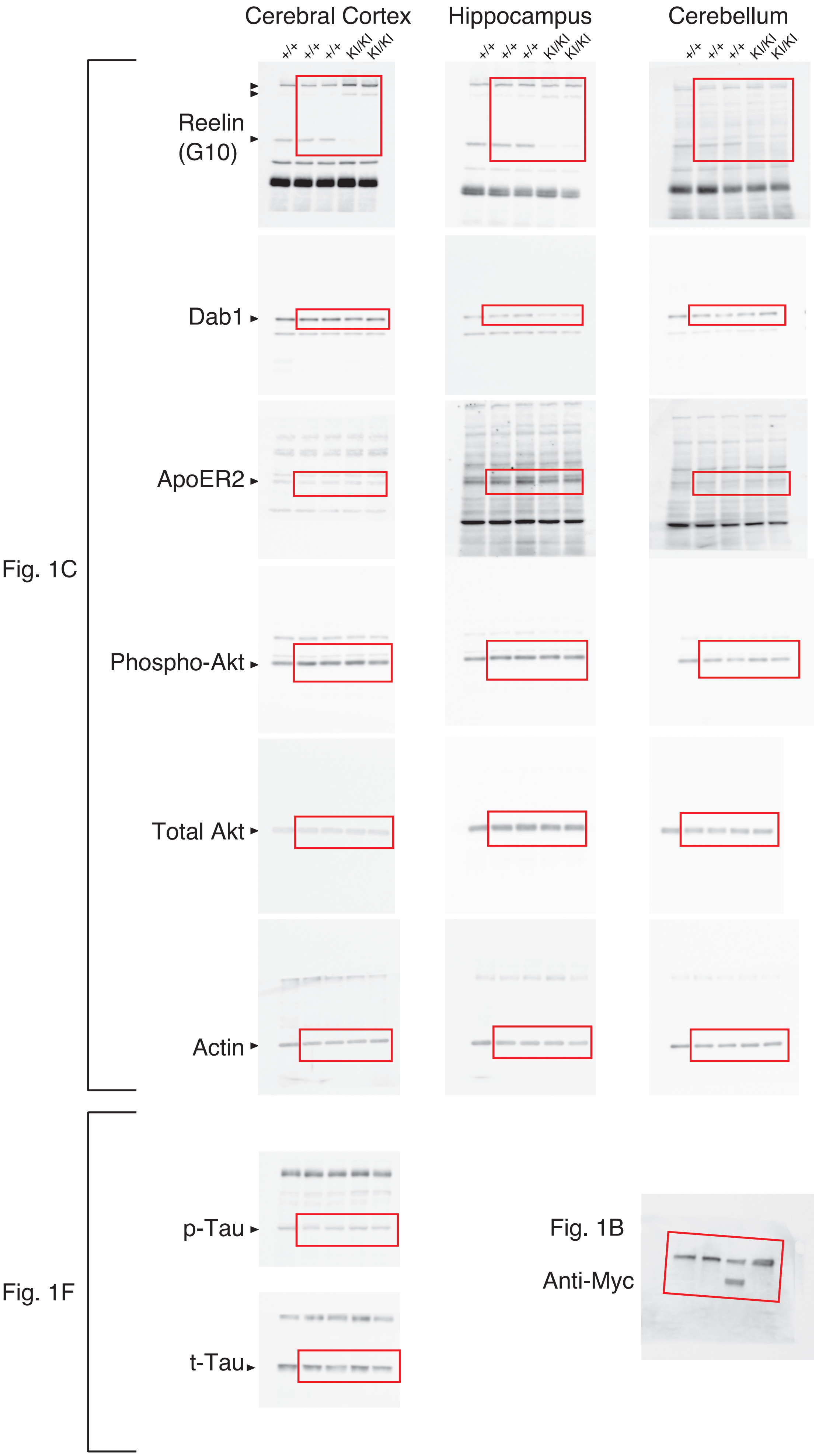

Supplemental Figure 1. The original uncropped images of Western blots for Figure 1. The left-most lanes are not used in Figure 1C and 1F.

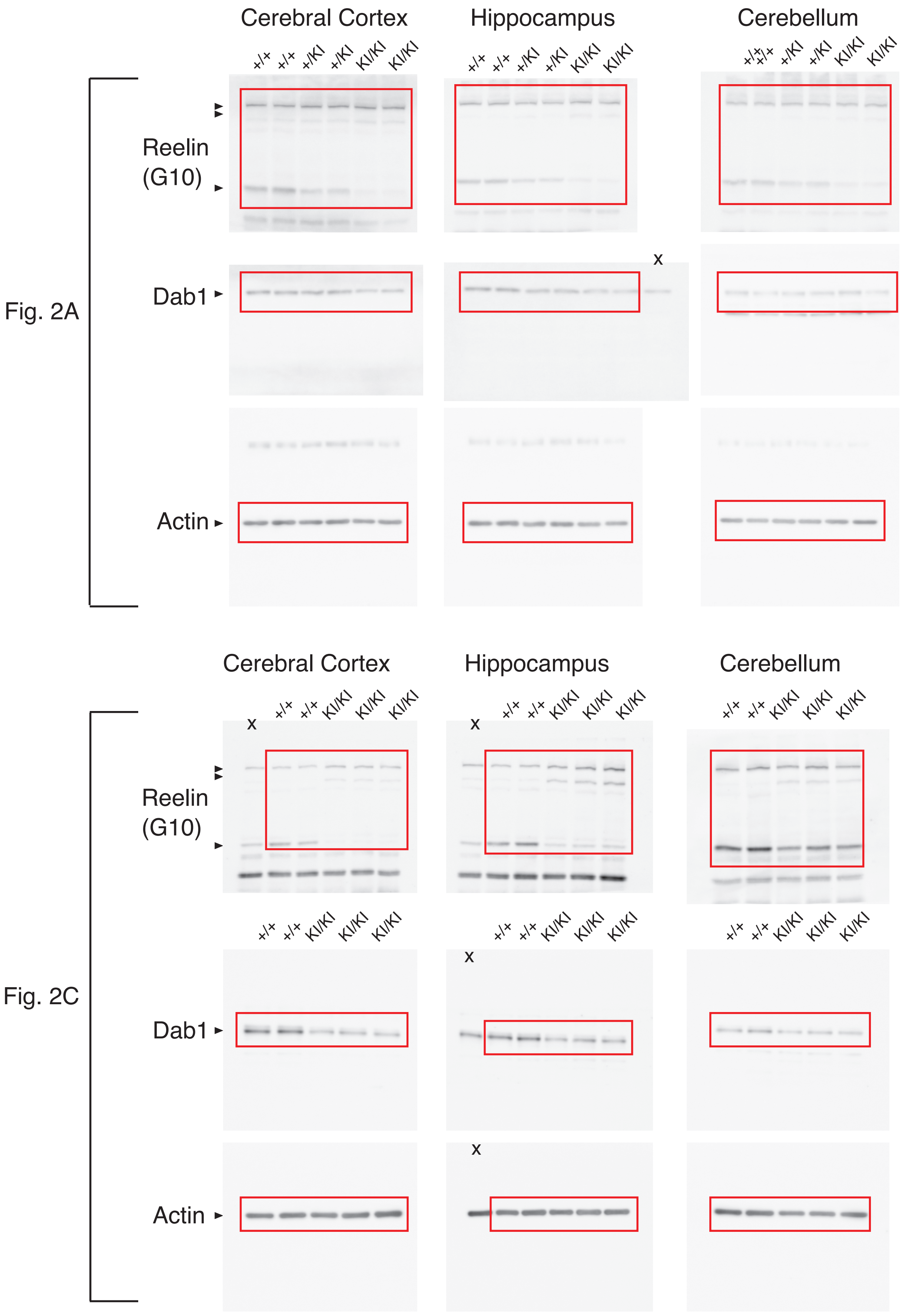

Supplemental Figure 2. The original uncropped images of Western blots for Figure 2.
The lanes indicated by x are dummies and not shown in Fig. 2.

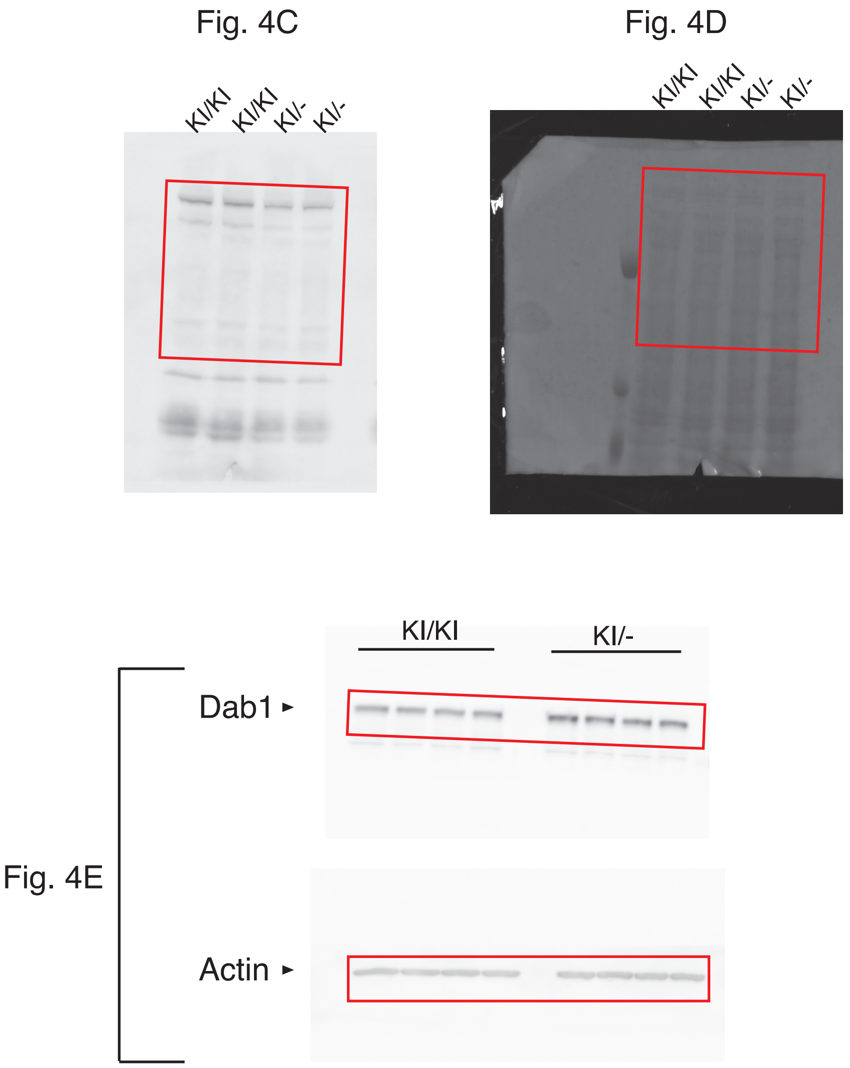

Supplemental Figure 4. The original uncropped images of Western blots for Figure 4.
